# Aqueous Cationic Fluorinated Polyurethane for Application in Novel UV-Curable Cathodic Electrodeposition Coatings

**DOI:** 10.3390/polym15183725

**Published:** 2023-09-11

**Authors:** Junhua Chen, Zhihao Zeng, Can Liu, Xuan Wang, Shiting Li, Feihua Ye, Chunsheng Li, Xiaoxiao Guan

**Affiliations:** 1 School of Environmental and Chemical Engineering, Zhaoqing University, Zhaoqing 526061, China; cehjchen@yeah.net (J.C.); zhihaozeng0203@163.com (Z.Z.); gmczunper@163.com (C.L.); pearships@163.com (X.W.); lishitingqc@163.com (S.L.); yefeihua@zqu.edu.cn (F.Y.); lichunsheng@zqu.edu.cn (C.L.); 2 Guangdong Provincial Key Laboratory of Environmental Health and Land Resource, College of Environmental and Chemical Engineering, Zhaoqing University, Zhaoqing 526061, China; 3 Green Fine Chemical Joint Laboratory, Qingyuan 511542, China; 4 China Electronic Product Reliability and Environmental Testing Research Institute, Guangzhou 511370, China

**Keywords:** cationic polyurethane, UV-curable, perfluoropolyether alcohol, cathodic electrodeposition coatings

## Abstract

Aqueous polyurethane is an environmentally friendly, low-cost, high-performance resin with good abrasion resistance and strong adhesion. Cationic aqueous polyurethane is limited in cathodic electrophoretic coatings due to its complicated preparation process and its poor stability and single performance after emulsification and dispersion. The introduction of perfluoropolyether alcohol (PFPE-OH) and light curing technology can effectively improve the stability of aqueous polyurethane emulsions, and thus enhance the functionality of coating films. In this paper, a new UV-curable fluorinated polyurethane-based cathodic electrophoretic coating was prepared using cationic polyurethane as a precursor, introducing PFPE-OH capping, and grafting hydroxyethyl methacrylate (HEMA). The results showed that the presence of perfluoropolyether alcohol in the structure affected the variation of the moisture content of the paint film after flash evaporation. Based on the emulsion particle size and morphology tests, it can be assumed that the fluorinated cationic polyurethane emulsion is a core–shell structure with hydrophobic ends encapsulated in the polymer and hydrophilic ends on the outer surface. After abrasion testing and baking, the fluorine atoms of the coating were found to increase from 8.89% to 27.34%. The static contact angle of the coating to water was 104.6 ± 3°, and the water droplets rolled off without traces, indicating that the coating is hydrophobic. The coating has excellent thermal stability and tensile properties. The coating also passed the tests of impact resistance, flexibility, adhesion, and resistance to chemical corrosion in extreme environments. This study provides a new idea for the construction of a new and efficient cathodic electrophoretic coating system, and also provides more areas for the promotion of cationic polyurethane to practical applications.

## 1. Introduction

Due to the unique microstructure produced between the hard and soft segments, polyurethane (PU) offers exceptional mechanical qualities. Nevertheless, the typical applications of solvent-based PU are constrained by environmental laws regarding the use of volatile organic compounds (VOCs) [1]. Traditional solvent-based PU is rapidly being replaced by environmentally friendly water-based PU as demand for environmentally friendly materials rises. A high-performance resin, known as waterborne polyurethane, utilizes water as a solvent in its formulation. In comparison to traditional solvent-based polyurethane, it offers superior properties such high hardness, wear resistance, strong adhesion, non-flammability, and low cost. It may also significantly lower the content of volatile organic compounds (VOCs) [2,3,4,5,6,7]. The textile, leather, furniture, paint, and construction industries all utilize it extensively [7,8,9,10,11]. Based on the nature of the ionization of hydrophilic groups in water, waterborne polyurethanes are classed as anionic, cationic, and nonionic. Ionic aqueous polyurethanes have been extensively investigated, and the anionic variety is now being produced commercially [12,13,14,15,16,17]. Cationic aqueous polyurethanes are typically synthesized by utilizing diols containing tertiary amine groups as chain extenders and quaternizing with alkylating agents or appropriate acids to produce water-soluble ionic groups. The mechanical properties of cationic aqueous polyurethanes that are not apparent in anionic materials are due to the capacity of the polar groups to form intermolecular hydrogen bonds with high intermolecular contact forces [18,19]. And cationic aqueous polyurethanes can be used for quaternary amination of the opposing ions contribute to hydrogen bonding and other ionic effects, but due to the complicated preparation process and poor stability after emulsification and dispersion, quaternary ammonium salt-based cationic aqueous polyurethanes are currently only sparingly studied [18,20,21,22,23,24].

Colloids known as cathodic electrophoretic coatings (EPD) are formed when charged particles and molecules that are suspended in a solvent move under the influence of an electric field and ultimately deposit on electrodes with opposing charges to create a dense surface film [25]. Cathodic electrophoretic coatings provide several advantages over traditional solvent-based coatings, including a straightforward procedure, high controllability, low cost, high permeability, electrochemical activity, and effective anti-corrosion properties [26,27,28]. Epoxy and acrylic cathodic electrophoretic coatings are actively being investigated. Through electrophoretic deposition of SH-SiO_2_ nanoparticles and resin binders, Zhang et al. created durable superhydrophobic SiO_2_ epoxy coatings with strong corrosion resistance but low weathering resistance [29].

Gong et al. created a cathodic electrophoretic solution utilizing cationic acrylate and EPD technology in order to create a conductive coating with high density and features that are hydrophobic and oleophobic [30]. The coating made using polyurethane electrophoresis solution in the traditional sense has relatively poor overall performance, such as corrosion and heat resistance, due to the introduction of hydrophilic groups, and further modification is needed for practical application in electrophoretic paints [31].

Fluorinated compounds are an excellent material for modifying. Liu et al. created a series of fluorinated aqueous polyurethane coatings by altering the content of fluorinated polymers to give them low surface energy and significant hydrophobic and oleophobic properties. The incorporation of C-F bonds can also augment the thermal stability and tensile properties of the coatings [32,33,34,35,36]. Unsaturated double bonds are added by the grafting of hydroxyethyl methacrylate (HEMA), which increases their thermal stability and offers active sites for light curing [37]. Finally, Liu et al. used a simple mixture of cationic aqueous polyurethanes and other UV-curing materials to prepare a new coating that improves its curing rate, reduces energy consumption, VOC emissions and reduces reactive diluents, and improves the overall physical properties of the coating, which can be applied to a variety of substrates, particularly heat-sensitive substrates [38,39,40,41,42,43].

In this experiment, cationic aqueous polyurethane was used as the electrophoresis fluid to introduce fluorinated polyether polyol (PFPE-OH) to provide low surface energy while grafting HEMA to provide a method to control the degree of cross-linking of the coating and improve the thermal stability of the coating. Finally, a novel UV-curable fluorinated cationic polyurethane-based cathodic electrophoretic coating was innovatively prepared using electrophoresis and UV curing technology. This study aims to investigate and evaluate the particle size, contact angle, water absorption rate, corrosion resistance, thermomechanical properties, and fundamental physical characteristics of the coating.

## 2. Experimental

### 2.1. Materials

Perfluoropolyether alcohol (PFPE-OH) was supplied by Hunan Nonferrous Chenzhou Fluorine Chemical Co. Ltd., Chenzhou, China. Isophorone diisocyanate (IPDI, >99.5%, NCO% ≥ 37.5%) was purchased from Bayer Chemicals (Leverkusen, Germany). Polycarbonate diol (PCDL, M_n_ = 1000), N-methyldiethanolamine (MDEA), trimethylolpropane (TMP), 1,4-butanediol (BDO), hydroxyethyl methacrylate (HEMA), 2-hydroxy-2-methyl-1-phenyl-1-propanone (Darocur 1173), glacial acetic acid (HAc), dibutyltin dilaurate (DBTDL) and 2,6-di-tert-butyl-p-cresol (DBHT) were purchased from Aladdin Reagent Co. Ltd., Shanghai, China. All organic solvents will be de-watered using 4 A° molecular sieve before use. All alcohols used were heated 120 °C in advance and de-watered under reduced pressure for about 2 h.

### 2.2. Cationic Aqueous Fluorinated Polyurethane Emulsion Preparation (WCFPU)

**Figure 1:**
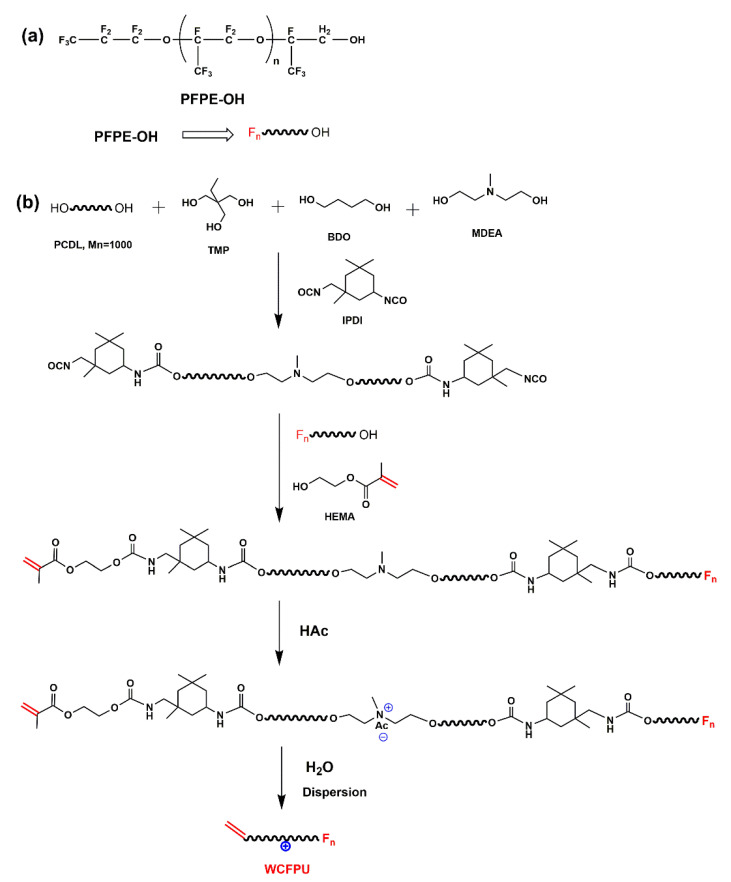
Waterborne cationic fluorinated polyurethane synthetic approaches (WCFPU). (**a**) The structural formula of perfluoropolyether alcohol (PFPE-OH); (**b**) the procedure for synthesis of modified polyurethane.

Fluorinated polyurethane polymer was synthesized via step-growth addition polymerization displayed in Figure 1. In a three-neck flask equipped with stirring paddle, thermometer, and nitrogen protection, PCDL and TMP were added after dehydration treatment, and then the temperature was increased to 80~85 °C. The temperature was then decreased to 60 °C after adding IPDI and DBTDL. BDO and MDEA butanone solution (40 wt%) were progressively added to extend the chain length and maintain the reaction for two hours. The molar functional group ratio of NCO/OH of the system at this stage was controlled to be 1.4. The system was adjusted with the proper quantity of butanone dilution when the viscosity became too high. The reaction was held for two hours to complete after the temperature was increased to 80 °C and PFPE-OH was introduced. The viscosity of the system was monitored at all times during the reaction, and butanone was added at any time. The temperature was then reduced to 60 °C. Butanone solution dissolved with HEMA and DBHT was slowly added dropwise, the remaining system NCO was capped with double bonding, and the prepolymer of CFPU was made by continuing the insulation for 2 h. The temperature was then reduced to 40 °C, and a small amount of HAc was added to neutralize the system for 30 min. Lastly, a small amount of deionized water was gently added to the prepolymer while the system was reverse-phase-emulsified at high speed for 30 min. Decompression at a negative pressure of −0.1 KPa and a temperature of 40 °C was used to remove the butanone solvent from the system. The final creamy white WCFPU emulsion with a solid content of 30 wt% was created.

### 2.3. UV-Cured Cathodic Electrodeposition Coating Preparation

A total of 30 wt% WCFPU emulsion was mixed with 5 wt% photoinitiator before being diluted in deionized water into a UV electrophoresis solution with a solid content of 15% and injected into the electrophoresis tank of the electrophoresis equipment. The tinplate was covered with the negative electrode, the pole plate was treated with phosphate, the tinplate was submerged in the electrophoresis tank, and there was a 10 cm space between the pole plates. The applied DC voltage was 60 V, and the electrophoresis period was 45 s. The cathode plate surface was then cleaned with a substantial volume of deionized water before being placed in an oven with a flash time of 3 min at 80 °C to eliminate the water. To achieve a uniform and smooth electrophoretic coating, the electrode plate was subjected to 100 mW/cm^2^ UV curing for a duration of 60 s.

### 2.4. Characterization

Fourier transform infrared spectra (FT-IR) were acquired using the KBr press method on a Bruker model VERTEX 70 spectrometer (Waltham, MA, USA), covering the wavelength range of 400–4000 cm^−1^. The hydrogen spectra (^1^H-NMR) of the compounds were obtained on a Bruker model AVANCE III HD 400 spectrometer with CDCl_3_ as solvents.

A volume of 0.1 mL of the sample was diluted with deionized water to a final volume of 50 mL and subjected to sonication for 30 min. The particle size distribution of the electrophoretic dispersion was determined by dynamic light scattering (DLS, Zetasizer Nano-ZS90, Malvern Instruments Ltd., Malvern, UK). A suitable amount of WCFPU emulsion was diluted 1000 times and sonicated for 30 min before a few drops of the dispersion were added onto a copper mesh using a pipette. The copper mesh was then dried in an infrared oven, followed by staining with a 5 wt% phosphotungstic acid solution for 5 min. Finally, the particle size morphology of the emulsion was examined using a transmission electron microscope (model JEM-100CX II from Japan Electronics, Beijing, China). The sample was coated with sputtered gold powder prior to examination under a scanning electron microscope (model SU-8220) capable of magnification ranging from 35 to 10,000 times.

Thermo Instruments ESCALAB 250 with XR-4 double anodes (Al/Mg) was used to perform X-ray photoelectron spectroscopy (XPS) studies under vacuum. The treated coated films were mounted on stainless steel with Cu tape on both sides and put in a sealed chamber at 10–8 mbar pressure overnight before being moved to the analytical chamber (pressure of 10–9 mbar) [2]. The XPS spectra of all materials were evaluated at a sampling depth of 6.6 nm and an electron emission angle of 45°, with the C1s line calibrated at 285.0 eV. A JC 2000C contact angle meter was used to measure the static contact angle of the coating, where the test droplets were water, diiodomethane, and hexadecane, respectively, and the volume of the droplets was 5 μL, and each sample was tested five times to take the average value. A homemade right-angled triangular test platform was designed so that the angle of the acute angle could be adjusted by lifting. The coated sample was placed on the hypotenuse of the right triangle, then the test droplet was placed on the sample, the angle was adjusted, and the slope angle when the droplet just started to move was observed and recorded as the rolling contact angle. The test droplets are water, diiodomethane and hexadecane, and the volume of the droplets is 30 μL, and each sample is tested three times to take the average value.

Differential scanning calorimetry (DSC) was used to evaluate the samples using a DSC 200F3 from Netzsch, Selb, Germany. In an aluminum sample tray, 10 mg of dried sample was sealed. To erase thermal history, the thermal cycle was conducted from 25–150 °C at a rate of 10 °C min^−1^ and maintained for 2 min. The glass transition temperature (Tg) and melting point (Tm) of polymers were then measured using a new thermal cycle that went from −70 to 250 °C at a rate of 10 °C min^−1^. A NETZSCH model TG209 was used to determine the thermal stability of UV-cured electrodeposited films. The test atmosphere was nitrogen and the thermal scan temperature range was 25 to 600 °C with a heating rate of 10 °C/ min. The thermomechanical characteristics of the coatings were tested using a dynamic thermomechanical analyzer model DMA 242C3 from NETZSCH, Germany. The greatest dynamic force was 2 N, the maximum static force was 0.5 N, and the maximum amplitude was 10 m. The test frequencies were 1.0 Hz, 3.333 Hz, and 5.0 Hz, with the temperature range being −50~100 °C. The temperature ramping rate was 5.0 K/min.

The tensile properties were assessed using a multifunctional electronic strength tester TS2000 (Beijing Chuangcheng Zijia Technology Co., Ltd., Beijing, China) at 10 mm/min, with test bars measuring 80 × 10 mm (length × width) and thicknesses ranging from 0.5 to 1.0 mm. Each measurement was repeated at least three times.

Three coated films with dimensions of 120 mm in length, 10 mm in width, and 0.5 mm in height were submerged in deionized water and kept at 25 °C room temperature for 72 h. Before weighing the samples, absorbent cotton paper was employed to remove the water from their surfaces. The equation below may be used to calculate how much water the samples absorbed [44]:

$$\;\mathrm{Absorption}\;\mathrm{percentage}=\frac{W_{2}-W_{1}}{W_{1}}\times 100\%$$

where *W*_2_ and *W*_1_ are the weight of the sample at 72 h water solubility and the weight of the original dry film, respectively.

The German QNIX 4200 (QNix, Koln, Germany) was used to gauge the coating’s thickness. The gloss of the coating at 60° was measured using an ETB-0686 gloss meter in accordance with the national standard GB 9754-88 [45]. The test was performed three times. Using a Faber Castell 9000 pencil, the hardness of the coating surface was evaluated in accordance with national standard GB/T6739-1996 [46]. QFH-HG600 was used to assess the adherence of the coating to the substrate in accordance with GB/T1720-1989 [47]. The impact resistance of the paint film surface was assessed using the impact tester QCJ-50 in line with national standard GB/T 1732-93 [48], the impact tester QCJ-50 was used to evaluate the impact resistance of the paint film surface. A Shanghai QTX tester was used to measure the painted board’s coating flexibility in line with GB/T 1731-93 [49]. The corrosion resistance of the electrophoretic coating was evaluated using four different solutions: 5.0 wt% NaCl, 0.5 mol/L CuSO_4_, H_2_SO_4_ (pH = 0, methyl orange staining), and NaOH (pH = 14, rhodamine staining). After 24 h, corrosion of the corresponding surfaces was seen after the addition of 0.5 mL drops to the coated and untreated areas, respectively.

## 3. Results and Discussion

**Figure 2:**
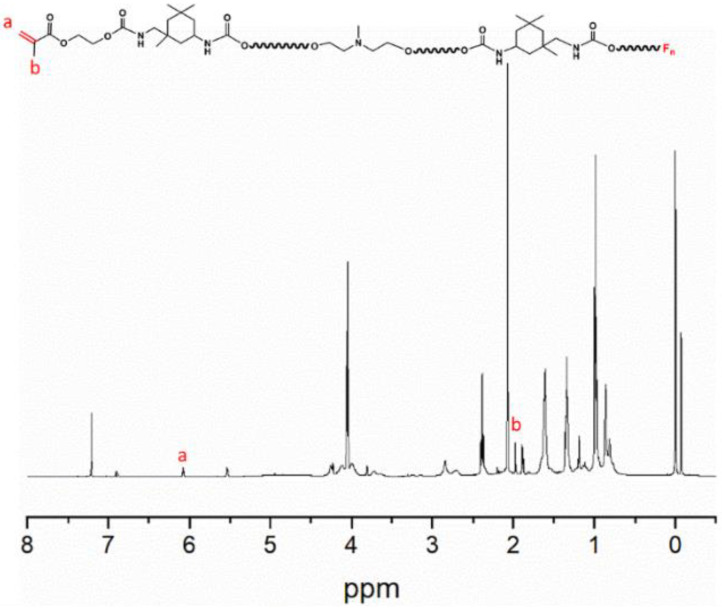
^1^H-NMR spectra of pure CFPU. The position of a is located at ~6.11 ppm weak peak is -C=C- bond, while the sharp peak of b at 2.01 ppm is due to -CH_3_ on the side group of the double bond.

**Figure 3:**
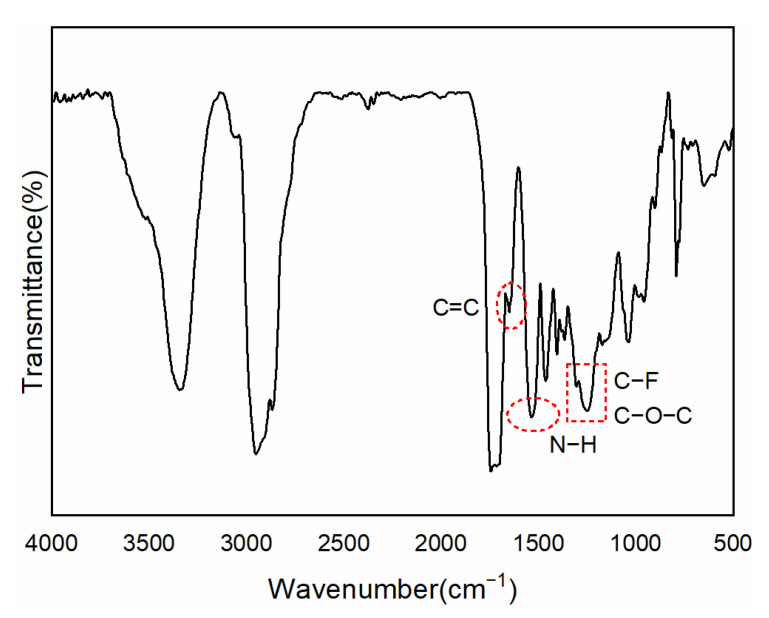
FT-IR spectrum of pure CFPU.

The IR spectrum of PFPE-OH is seen in Figure S1. The distinctive absorption of the alcohol hydroxyl group was shown by the wide peak at 3412.31 cm^−1^, while the C-O-C and C-F characteristic absorption peaks were located between 1000 and 1400 cm^−1^. Among these, the distinctive absorption peak of -CF_3_ was 1232.62 cm^−1^, whereas that of -CF_2_ was 1123.58 cm^−1^. The nuclear magnetogram of CFPU, which had a prior pretreatment to get rid of the organic solvent, is shown in Figure 2. The -C=C- bond was represented by the faint peak at 6.11 ppm, while the -CH_3_ on the double bond side group was represented by the sharp peak at 2.01 ppm. To verify the success of double bond grafting and the presence of fluorinated monomers in the system, the IR spectra of CFPU are shown in Figure 3, and it is evident that it has a faint peak at 1635.4 cm^−1^ that is indicative of unsaturated double bond stretching vibration absorption. Also visible are the distinctive absorption peaks of C-O-C and C-F at 1100–1350 cm^−1^. The medium broad peak at 3321 cm^−1^ is the N-H stretching vibration. The splitting peaks at 2939 cm^−1^ and 2864 cm^−1^ correspond to the stretching vibrations of -CH_3_ and -CH_2_-, respectively, which are mainly the methylene absorption peaks on the cationic chain extender (MDEA) and polyester structure. A strong and sharp characteristic peak at 1729 cm^−1^ is the stretching vibration of C=O. The characteristic carbamate N-H bending vibration absorption peak at was 1541 cm^−1^. The FPU prepolymer was effectively produced, as seen by the IR and NMR pictures, and the unsaturated double bond serves as the active site for light curing. As a result, the ATR measurement of the coated film following UV electrophoresis demonstrated in Figure S2 that the unsaturated double bond at 1635.4 cm^−1^ vanishes, indicating that WCFPU has fully cured.

**Figure 4:**
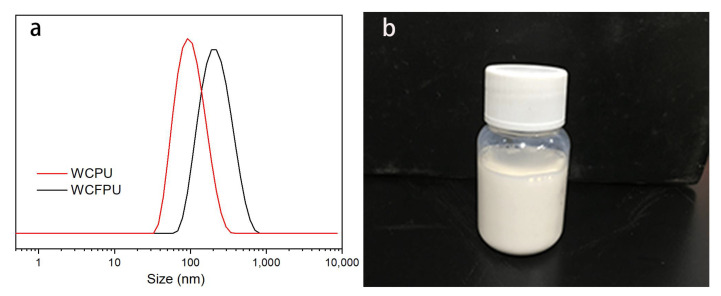
The DLS trace of WCFPU emulsion (**a**) and the corresponding picture of the latex (**b**).

Figure 4a shows the average particle size distribution of WCFPU emulsions, which is around 91.3 nm smaller than that of the usual WCPU non-fluorinated emulsion. This indicates that the particle size of polyurethane emulsions modified with fluorinated polyether alcohols increased, and their hydrophobic ends were encapsulated within the polymer, while the hydrophilic chain segments of cationic tertiary amine groups were dispersed on the surface of the macromolecule, forming a core–shell-like structure, so their particle size became larger.

**Figure 5:**
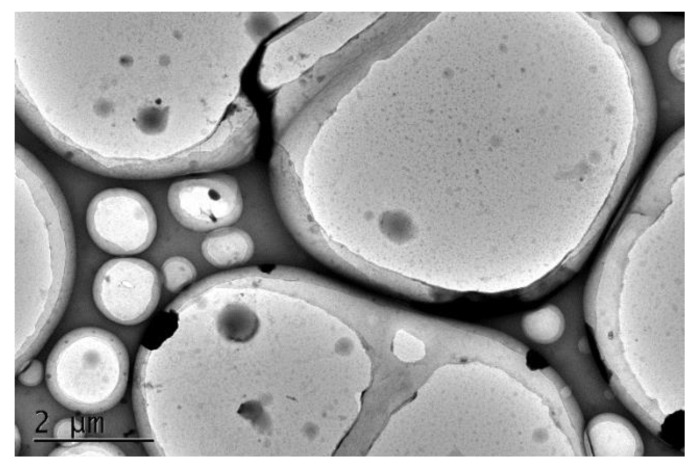
TEM image of WCFPU dispersions.

**Figure 6:**
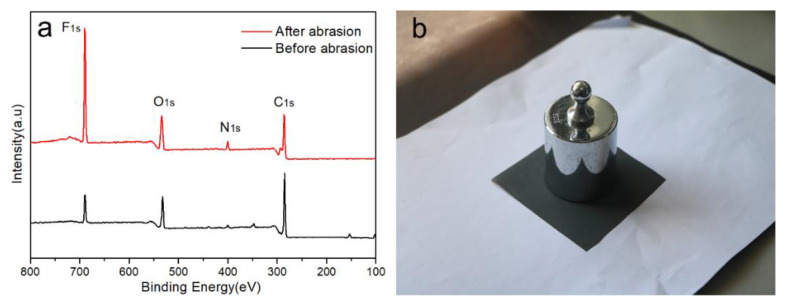
XPS spectra of UV-curable EPD films before and after the abrasion test (**a**) and corresponding self-made friction device (**b**).

The analysis of the WCFPU latex particle morphology is shown in Figure 5 and is enlarged to a viewing distance of 2 μm. The photos show that the grain size determined in Figure 6 is equivalent to the WCFPU latex grains, which are rather homogenous round spheres with a grain diameter of around 200 nm. The outside dark half was most likely a chain segment of an aqueous cationic chain extender, whereas the inner brilliant piece was most likely the fluorinated polyurethane chain segment with a low electron cloud density [50]. The thickness of the shell layer is about 19 nm. The photographs also further illustrate the nucleoshell structure with hydrophilic as the shell and hydrophobic as the nucleus.

Figure S3 show the results of an investigation into the surface morphology of the electrophoretic coating. Each area had a uniform shape and a smooth surface. It demonstrated that the system was evenly distributed, that there was no macroscopic phase separation, and that during UV curing, a highly cross-linked network formed.

To understand the fluorine chain segment movement, the XPS test was utilized to examine the surface chemistry of the UV-curable electrophoresis film. The films were polished with 500 g weights for 50 cycles, and then baked in an oven at 140 °C for 30 min. The XPS spectra before and after the coating rubbing are shown in Figure 6. It showed that the electrophoretic coating was mostly composed of C, N, O, and F as the main four elements. The fluorine atom signal intensity of the sanded and baked adhesive coating was greater than that of the unpolished one, indicating that fluorine-containing chain segments with low surface energy were more likely to migrate to and enrich the surface of the film/air. This resulted in a flat coating with a specified amount of hydrophobicity and decreased the interfacial energy on the surface of electrophoretic coating. Table S1 displays the mass percentages of each atom as determined by XPS measurements.

The Table S1 demonstrated that the fluorine atom content increased from 9.89% before sanding to 27.34% after sanding and baking, whereas the predicted average fluorine atom concentration is 4.82%. It implied that fluorine-containing chain segments with low surface energies moved to the surface of coating preferentially and were enriched during UV curing film production. The moderate amount of heat facilitated polymer chain segment migration and hydrophobic properties.

**Figure 7:**
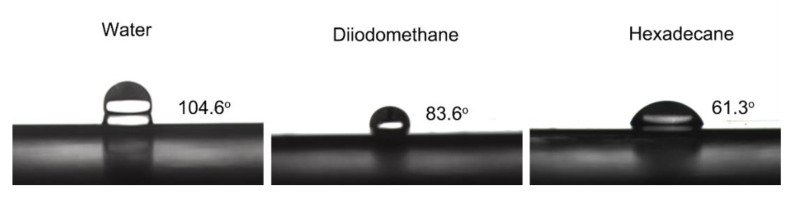
Images of droplets and the contact angles of water, diiodomethane, and hexadecane on the coating.

We examined the static and rolling contact angles of water, diiodomethane, and hexadecane droplets on the surface of the coating to assess the wettability of the fluorinated cationic polyurethane electrodeposition coating. The corresponding surface tensions of droplets were found to be 72 mN·m^−1^, 50 mN·m^−1^, and 27 mN·m^−1^ [51]. As shown in Figure 7, the static contact angles of droplets in 5 mL on the coating and the instantaneous contact angles of these droplets on the coated surface are 104.6 ± 3°, 83.6 ± 2°, and 61.3 ± 1°, respectively. This resulted from UV-curing the fluorine-containing long-chain segments, which improved the resistance of coating to water and solvents as well as the formation of regular, low-surface-energy liquid crystal forms on the surface of the latex film.

Droplets of water, diiodomethane, and cetane rolling on the coating in a volume of 50 mL are shown in Table 1, as well as their contact angles. Water droplets rolled off the surface neatly; although they had a smaller rolling angle, dii-odomethane and cetane tended to leave traces and did poorly as anti-fouling agents against oil-based pens. This was mainly due to the poor compatibility of the fluorine-containing chain segments in the coating resin structure, resulting in reduced film denseness, and the relatively insufficient content of CF_3_ groups on the coating surface. The electrophoretic coating film appeared to have excellent hydrophobicity, which was consistent with the XPS test results. The average water absorption of our test coating was 2.82%, and the surface layer was enriched with many fluorine-containing chain segments, which lowered its surface energy.

**Figure 8:**
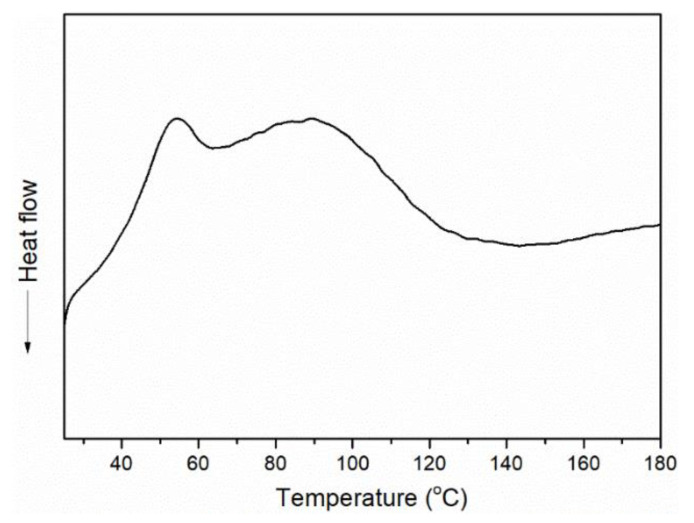
DSC curves of UV-curable EPD coatings.

Because of the higher organofluorine polymer content and the addition of C-F polar groups, as well as the increasing relative molecular mass of the polymer (Figure 8 show the DSC curves of UV cured electrodeposition coatings), the glass transition temperature of the soft segment of polyurethane in WCFPU was 53.9 °C. The figure indicated that the higher glass transition temperature, which was between 89 and 120 °C [52], which caused by the carbamate group of the hard chain section. It can be clearly observed from the figure that, as with the Tg of the soft segment, the Tg and hydrogen-bonding interactions of the hard segment decreased with the increasing length of the (-CH_2_-CH_2_-O-)_n_ chain segment, and there was no significant phase separation.

**Figure 9:**
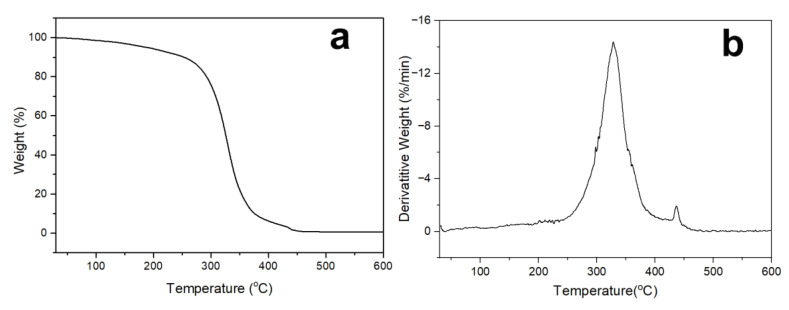
TGA curves (**a**) and their corresponding DTG curves (**b**) of UV-curable EPD coatings.

The thermogravimetric curves of UV-cured electrophoretic coatings are shown in Figure 9. At a temperature of about 143 °C, the coating started to break down, as seen in the photograph, which was probably the result of minute molecules dissolving within the system. The carbamate structure in the structure gradually disintegrated during the second breakdown stage, which took place between 250 and 380 °C [53,54]. Between 428 and 452 °C, the breakdown entered its third stage. The improvement in the thermal stability of the UV-cured electrophoretic coating was due to the inclusion of C-F with higher bond energies, the creation of shielding protection for the interior of the polymer, and the rise in cross-link density following double bond curing.

**Figure 10:**
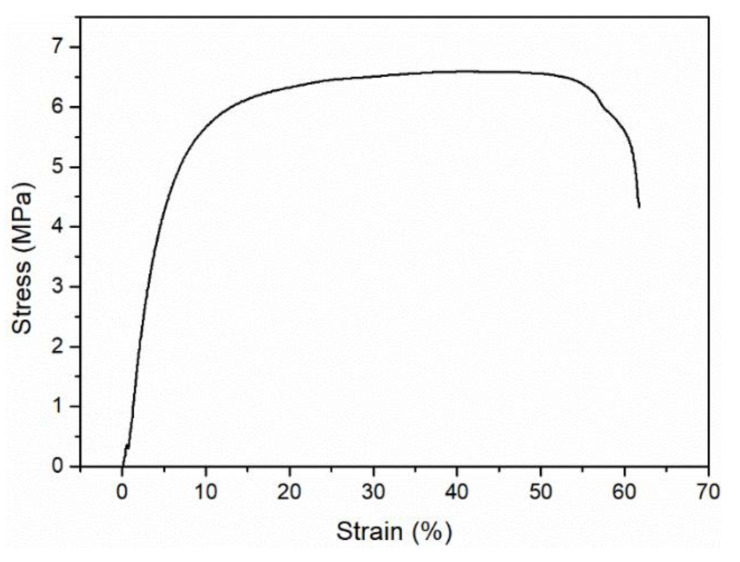
Stress–strain curves of UV-curable EPD coatings.

The stress–strain curve for the UV electrodeposition coating is shown in Figure 10. According to this curve, the fluorinated polyurethane-coated film had a tensile strength of 6.59 MPa and an elongation at break of 61.11%. The tensile strength of the original fluorine-free polyurethane coating sample was 5.68 MPa and the elongation at break was 20.14%. PCDL was used as polyester in the WCFPU structure because it provided good tensile properties. If a smaller amount of PFPE-OH was added, it led to an increase in the number of fluorinated groups introduced into the hard chain segments of the polymer, which further affected the increase in tensile strength and decrease in elongation at the break of the coated film. Furthermore, the strain-hardening phenomenon became more pronounced when fluorinated groups were introduced, which enhanced the tensile strength and reduced the flexibility of the electrophoretic film. The perfluorinated long-chain structure of PFPE-OH imparted a high degree of hydrophobicity to the membrane, increasing the content of fluorinated groups and the ratio of hydrophobic to hydrophilic chain segments, thus increasing the particle size of the emulsion and decreasing the density variation of the surface. The increase in emulsion particle size would lead to a decrease in the denseness of the emulsion after film formation, and therefore a decrease in mechanical properties [55].

**Figure 11:**
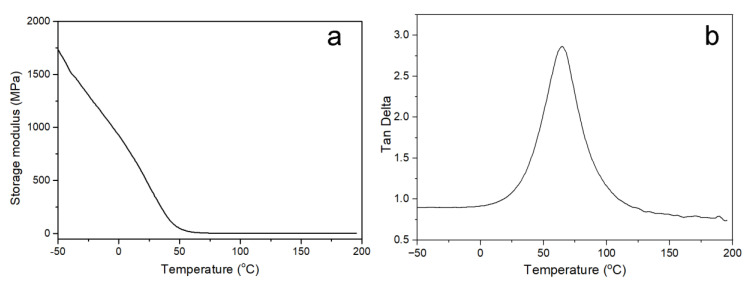
Temperature dependence of storage modulus (**a**) and tan δ (**b**) for UV-curable EPD films.

The thermomechanical behavior of the UV-curable EPD coatings is depicted in Figure 11, and the figure illustrates the great compatibility of the polyurethane system. If there was no splitting, one tan δ appeared on the graph, and conversely, two tan δ appeared if the structure was split. It can be observed from the graph that the energy storage modulus of the coating decreased with increasing temperature. The glass transition temperature (Tg) of the soft section corresponded to 64.7 °C, while the glass transition temperature of the hard section was less pronounced. Generally, the higher the glass transition temperature, the more easily molecular chain movement was hindered and the more rigid the polymer. The internal trifunctional TMP cross-linking site that restricted the mobility of the molecular chain, along with hydrogen bonding and the formation of a cross-linked network in the structure, was most likely to blame for the 2.84 damping factor.

**Figure 12:**
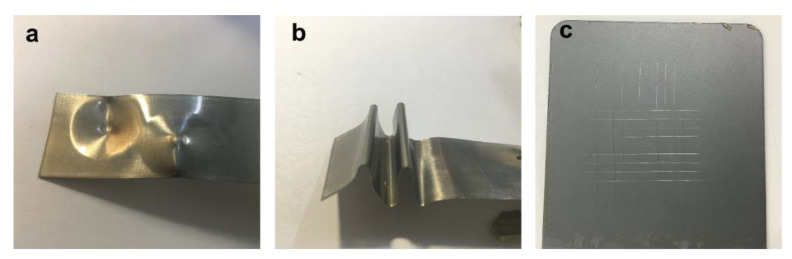
Impact resistance (**a**), flexibility (**b**), and adhesion (**c**) test on the UV-curable EPD coatings.

The UV electrodeposition coating offered high physical qualities as well as good hydrophobicity and thermal stability. As seen in Figure 12a, when the coated tinplate was struck with a 1000 g impact hammer from a height of 50 cm in both the forward and backward directions, the surface did not break or peel off. In Figure 12b, the test plate was bent 180° with a rod of 1 mm in diameter, and the coated surface did not crack at any point. Figure 12c shows the results of the adhesion test. The adhesion test uses the scratch technique described in GB/T 9286-1998 [56]. There are six degrees of adhesion, with level 5 being the worst. From level 0 to level 5, coating adherence diminishes. According to Figure 12c, the modified coating has a 0–1 level of adherence. The scribed section did not come off when the 3M tape was removed, suggesting strong adherence. This was due to the use of polycarbonate diol (PCDL) as an excellent polyester raw material for polyurethane light-curing coatings, providing excellent flexibility, adhesion, abrasion resistance, and chemical resistance to the coating.

**Figure 13:**
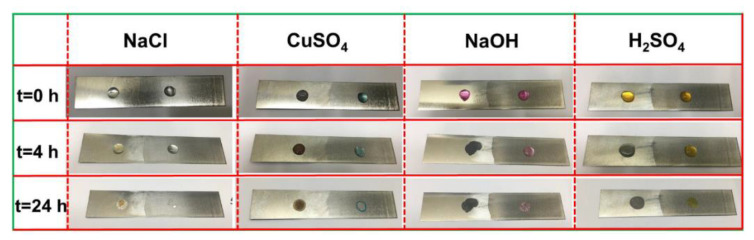
Effect of salt solution, strong acid solution, strong alkali solution, and corrosion cell on surface of the coatings.

Figure 13 shows the chemical corrosion of the electrophoretic coating surface using different 5.0 wt% NaCl solution, 0.5 mol/L CuSO_4_ solution, H_2_SO_4_ solution (pH = 0, stained with methyl orange), and NaOH solution (pH = 14, stained with rhodamine) for different times. The droplets shrunk on the coated surface, while on the uncoated surface, they were spread flat. We found that when the polished uncoated tinplate came into touch with the copper sulfate solution, it immediately became covered in copper due to primary cell corrosion. After 4 h, due to partial evaporation of water, the volume of liquid was reduced and the surface of the coating appeared without corrosion or damage, but these liquids had caused varying degrees of corrosion to the uncoated tinplate. After 24 h, the water evaporated completely and the surface of the coating remained intact, while the uncoated surface showed severe chemical corrosion. It was indicated that the coating had good corrosion resistance and great potential for application in various corrosion-resistant industries.

## 4. Conclusions

In this study, a terminal fluoroalkyl cationic unsaturated polyurethane emulsion (WCFPU) was made by introducing fluorinated polyether polyol (PFPE-OH) as a side chain and grafting hydroxyethyl methacrylate (HEMA) to provide unsaturated double bonds to IPDI and polyol, and then dispersed and prepared into UV electrophoretic coatings with a solid content of 15%. The average particle size of the WCFPU emulsion was found to be about 190 nm, while the particle size of the normal non-fluorinated WCPU was 91.3 nm. Observing the TEM of the emulsion particles, we could speculate that the fluorinated cationic polyurethane emulsion was a core–shell structure with hydrophobic ends encapsulated within the polymer and hydrophilic ends on the outer surface. After wear testing and baking, it was found that the fluorine atom content of the coating increased abruptly from 8.89 to 27.34%. The static contact angle of the coating to water was 104.6 ± 3°, and the water droplets rolled off without traces, indicating that the coating had very good hydrophobicity. This UV electrophoretic coating had excellent thermal stability and tensile properties, and its thermomechanical behavior indicated a high polymer crosslink density. The coating also underwent the tests of impact resistance, flexibility, adhesion, and resistance to chemical corrosion in extreme environments.

## Figures and Tables

**Table 1:** Sliding angle of water, diiodomethane, and hexadecane droplets for different volumes on the coating.

Test Droplets	Sliding Angle (50 μL)
Water	37.8 ± 1.5°
Diiodomethane	11.2 ± 0.5°
Hexadecane	9.7 ± 0.4°

## Data Availability

Not available.

## Supplementary Materials

The following supporting information can be downloaded at: https://www.mdpi.com/article/10.3390/polym15183725/s1.
