# Supplementary material for: Aqueous Cationic Fluorinated Polyurethane for Application in Novel UV-Curable Cathodic Electrodeposition Coatings
Source: Polymers (Basel). 2023 Sep 11;15(18):3725. doi: 10.3390/polym15183725 (PMC10535655; doi:10.3390/polym15183725)
Supplement: Supplementary file 1 [file polymers-15-03725-s001.zip › polymers-2494751-supplementary.pdf]

Aqueous cationic fluorinated polyurethane for application in novel UV-curable cathodic electrodeposition coatings

Junhua Chen [1,2,3], Zhihao Zeng [1], Can Liu [1], Xuan Wang [1], Shiting Li [1], Feihua Ye [1,2,3], Chunsheng Li [1,2,3] and Xiaoxiao Guan [4,*]

[1] School of Environmental and Chemical Engineering, Zhaoqing University, Zhaoqing 526061, China; cehjchen@yeah.net (J.C.); zhihaozeng0203@163.com (Z.Z.); gmczunper@163.com (C.L.); pearships@163.com (X.W.); lishitingqc@163.com (S.L.); yefeihua@zqu.edu.cn (F.Y.); lichunsheng@zqu.edu.cn (C.L.)

[2] Guangdong Provincial Key Laboratory of Environmental Health and Land Resource, College of Environmental and Chemical Engineering, Zhaoqing University, Zhaoqing 526061, China

[3] Green Fine Chemical Joint Laboratory, Qingyuan 511542, China

[4] Zhaoqing Institute of Economic, Social and Historical Culture, Zhaoqing University, Zhaoqing 526061, China

[*] Correspondence: shenxiao0601@163.com

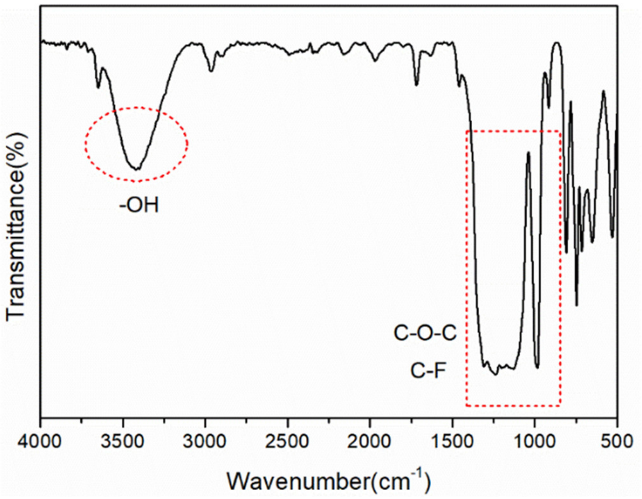

Figure S1 FT-IR spectrum of pure PFPE-OH

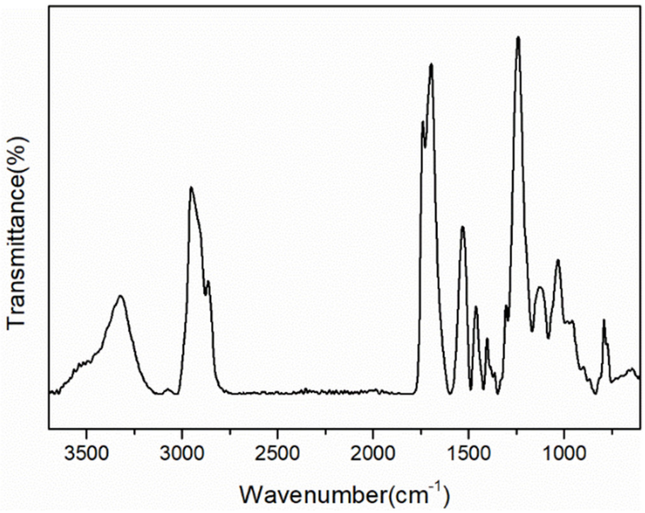

Figure S2 FT-IR spectrum of UV-cured film

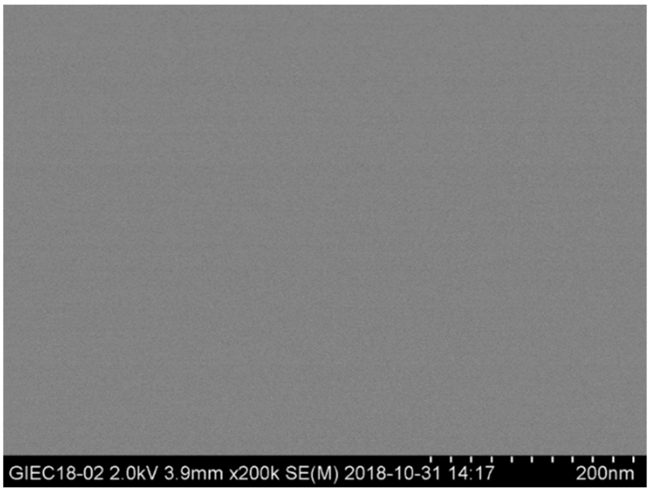

Figure S3 SEM photos of UV-curable EPD films

Table S1 Atomic compositions of UV-curable EPD films before and after the abrasion test

Atom	Atom % Before Wear	Atom % After Wear	Atom %* Ave. in Coating
F	9.89	27.34	4.82
C	70.16	51.38	71.34
O	17.15	16.85	19.26
N	2.8	4.43	3.58
